# What We Know and Do Not Yet Know About the Canine Model of Lymphoma in Human Medicine—The Current State of Knowledge

**DOI:** 10.3390/cancers17040596

**Published:** 2025-02-10

**Authors:** Daria Będkowska, Sara Al-Ameri, Agnieszka Wieczorek, Joanna Bubak, Marta Miszczak

**Affiliations:** 1 EZA Student Science Club, Department of Epizootiology and Clinic of Birds and Exotic Animals, Division of Infectious Diseases and Veterinary Administration, The Faculty of Veterinary Medicine, Wrocław University of Environmental and Life Sciences, Grunwaldzki Sq. 45, 50-366 Wrocław, Poland; 118245@student.upwr.edu.pl (D.B.); 118225@student.upwr.edu.pl (S.A.-A.); 115786@student.upwr.edu.pl (A.W.); 2 Department of Pathology, Division of Pathomorphology and Veterinary Forensics, The Faculty of Veterinary Medicine in Wrocław, Wrocław University of Environmental and Life Sciences, 31 Norwida St., 50-375 Wrocław, Poland; joanna.bubak@upwr.edu.pl; 3 Department of Epizootiology and Clinic of Birds and Exotic Animals, Division of Infectious Diseases and Veterinary Administration, The Faculty of Veterinary Medicine in Wroclaw, Wrocław University of Environmental and Life Sciences, Grunwaldzki Sq. 45, 50-366 Wrocław, Poland

**Keywords:** lymphoma, cancer, dog, human, non-Hodgkin’s lymphoma, comparative oncology

## Abstract

This review comprehensively compares lymphoma in humans and dogs, highlighting the canine model’s utility in translational research. Canine lymphoma (cL), predominantly diffuse large B-cell lymphoma (DLBCL), mirrors human non-Hodgkin’s lymphoma (NHL) in its clinical presentation, including lymphadenopathy, systemic symptoms (e.g., fever, weight loss), and hematological abnormalities. Morphologically, cL and NHL share similarities in DLBCL subtypes (centroblastic, immunoblastic, anaplastic), although some variations exist, such as the presence of macronuclear medium-sized cells in canine polymorphonuclear centroblastic lymphoma, not observed in humans. Canine and human lymphomas share molecular mechanisms, including the activation of key pathways like NF-κB and mTOR, and genetic and epigenetic alterations. The tumor microenvironment influences tumor growth and immune evasion in both species. Both species exhibit similar responses to chemotherapy, primarily CHOP-based protocols, although canine lymphoma often progresses more rapidly, offering advantages for shorter clinical trials. Molecular targeted therapy is emerging as a promising treatment, with human therapies like rituximab and chimeric antigen receptor T-cell therapy showing efficacy, and canine treatments still developing. Epidemiological data reveal overlapping risk factors, including exposure to environmental carcinogens (e.g., household chemicals, pollution) and the potential influence of sex hormones, although the role of sex hormones requires further investigation in canines. While staging systems differ slightly (Lugano modification of Ann Arbor for humans, WHO system for dogs), both consider disease extent and systemic involvement. Prognostic factors, such as lactate dehydrogenase (LDH) levels, are relevant in human NHL but have not shown consistent utility in cL. This study concludes that the spontaneous development of cL in immunocompetent dogs, coupled with its clinical, histological, and therapeutic similarities to human NHL, makes the canine model invaluable for preclinical research, accelerating the development of novel diagnostic tools and therapies for both human and canine lymphoma. The shared environmental risk factors and shorter disease progression in dogs further enhance the translational potential of this model, promoting a One Health approach to cancer research.

## Simple Summary

Canine lymphoma (cL) is currently one of the most common hematopoietic cancers in dogs and shares many similarities with non-Hodgkin’s lymphoma (NHL) in humans. The aim of this article was to assess the potential of these analogies that make cL a suitable model for studying NHL and create a capability in understanding of causes, progression, and therapies for both species. This makes canine lymphoma an excellent model for developing new immunotherapeutic approaches in translational oncology research.

## 1. Introduction

Lymphoma is the most common neoplasm originating from hematopoietic tissue occurring in dogs, usually detected in advanced stages (III–V) [1]. The classification used in human medicine, taken from the World Health Organization (WHO) Classification for Tumors of Lymphoid Tissues [2], is applicable for diagnosis, where it is simultaneously observed that in both humans and dogs, the diffuse large B-cell type (DLBC) is the most common [3]. The dog appears to be a suitable animal model for lymphoma, given the clinical, genetic and histopathological similarities in the course of the disease [3]. An additional aspect is the often more rapid course of the disease in dogs [4]. Clinical trials are shorter in duration, as are periods of remission, and dogs often share a similar living environment to their caregivers, allowing observation of the influence of environmental factors on the development of lymphoma [1]. The unique status of dogs in societies means that they are treated like family members, making their caregivers want to allow them access to quality medical care [5]. In dogs, lymphoma develops spontaneously; there is also greater similarity in tumor gene sequences [6], and development better illustrates the natural course of the disease than in other animal models, such as mice [1]. Because of the similarity of gene mutations found in humans and dogs, responses to therapy in both cases are similar [7,8]. The purpose of this article was to provide a comprehensive comparative analysis of lymphoma in humans and dogs consistent with the current state of knowledge. Available information was collected and organized, creating a broad overview of the many aspects of similarities and differences, proving the preponderance of the canine model over other animal models. Given that lymphoma poses a real threat to the health and life of both species, deepening our knowledge of them may contribute to improvements in their diagnosis and treatment [5]. By understanding the mechanisms of the immune response, the causal relationships leading to the onset and progression of the disease, it will be possible in the future to introduce innovative techniques in clinical practice [7].

## 2. Epidemiology and Risk Factors for Canine and Human Lymphomas

Lymphoma in humans is a diverse group of hematologic neoplasms [8]. Non-Hodgkin’s lymphoma (NHL), the most common hematological malignancy worldwide, is a blood cancer affecting the lymphatic system [9]. It is the 11th most common cancer diagnosis and the 11th leading cause of cancer death globally [10]. The incidence rate of NHL was 18.6 per 100,000 men and women annually, with a mortality rate of 5.0 per 100,000 per year. These figures are based on data from 2017–2021 for new cases and 2018–2022 for deaths [10]. The incidence of NHL has been rising, with significant regional variations. For instance, Australia and New Zealand have seen notable increases in incidence rates, while North African countries experience higher mortality rates [11]. According to the Global Burden of Diseases report (2019), China faces a significant and increasing burden of lymphoma, with approximately 101,500 new cases and 47,000 deaths annually [12]. In the United States, the incidence rate (per 100,000) doubled from 1975 to 2007 (21.3) and then stabilized, while the 5-year relative survival rates improved from 47% in 1975 to 73.8% in 2018 [13]. What is noteworthy, in contrast with many European and North American countries, is that there has been a steady and substantial increase in the incidence of NHL in England over the past four decades. Overall, the highest increase was observed in males (115%) and those aged 65+ years (109%) [14]. Projections indicate that by 2040, the number of new NHL cases could reach around 778,000 annually [11]. Risk factors such as obesity and HIV infection are positively correlated with NHL incidence. NHL occurs more frequently in men, individuals over 65 years of age, and those with autoimmune disorders or genetic determinants [11]. Infectious agents, primarily viruses, and contact with chemical substances have also been identified as risk factors for the disease [11]. Most NHL malignancies originate from mature B lymphocytes, with fewer cases arising from T lymphocytes or natural killer (NK) cells [15]. The prevalence of NHL subtypes varies geographically; in Western countries, approximately 85% of cases are B-cell lymphomas and about 15% are T-cell lymphomas, though these proportions often differ across regions [16]. Developing regions, on the other hand, report more cases of T- and NK-cell lymphoma and a lower frequency of B-cell lymphoma, with significantly higher occurrences of high-grade B-cell lymphoma (59.6%) compared to Western countries (39.2%) [17].

In dogs, lymphoma (canine lymphoma, cL) is one of the most common hematopoietic malignancies, ranking as the third most frequently diagnosed tumor in small animal veterinary practice [18]. cL is more common in dogs over 10 years of age and in medium to larger breeds, with over 80% of cases diagnosed as the multicentric form, predominantly diffuse large B-cell lymphoma [19,20]. Epidemiological studies suggest dogs can be valuable models for understanding human health risks due to their shared environment and similar exposure to risk factors [21]. Although no definitive cause for cL has been identified, living in industrial areas and contact with carcinogens, such as household chemicals, pollution, and magnetic fields, are known to increase the risk of disease [22].

The etiology of lymphoma in both humans and dogs is multifactorial, involving viral infections, genetic and molecular predispositions, immunosuppression, and environmental influences [8]. Humans and dogs share the same living spaces, breathe the same air, and their health is often influenced by their shared socioeconomic conditions, including access to medical or veterinary care, housing quality, and diet [4]. Common environmental risk factors are evident in the geographic distribution of lymphoma subtypes in both species [21,23]. For instance, cL prevalence is higher in dogs living in high-traffic or industrial areas [24,25], paralleling the increased incidence of NHL in humans exposed to benzene, a significant component of vehicle emissions [26]. Moreover, exposure to secondhand smoke in dogs’ homes is linked to a higher risk of cL [22], mirroring the increased risk of follicular NHL in non-smokers exposed to passive smoking [27].

Another known risk factor for developing these cancers is the effects of sex hormones. In human oncology, a correlation between the amount of sex hormones and the development of lymphoma has been observed for years [28,29]. It is suggested that female sex hormones (estrogens and progesterone) may protect against NHL, as it occurs less frequently in women than in men, and its incidence increases after menopause. Females using oral hormone therapy, as well as those undergoing hormone therapy post-menopause, exhibited a reduced risk of developing non-Hodgkin’s lymphoma [30,31]. There is evidence of the influence of sex hormones on the development of tumors in dogs as well. For example, according to [32], spayed females before their first estrus are less likely to develop mammary gland tumors [32]. In subsequent years, ovariohysterectomy continued to be associated with an increased risk of lymphoma [33]. Among the 30 breeds with adequate sample sizes for analysis, 23 exhibited an elevated risk associated with neutering. Specifically, neutering was linked to an increased risk of lymphoid neoplasia in males in 20 breeds and in females in 17 breeds. No breeds demonstrated a reduced risk of lymphoid neoplasia in neutered animals [34]. Although, there are reports in which this association has not been substantiated [35].

While there is not a global database specifically tracking canine lymphoma, studies tend to focus on specific regions. In the United States, cL is one of the most frequently diagnosed cancers in dogs [36]. Studies from Europe and Australia also report relatively high incidences, although the disease appears to be more common in certain breeds, such as Boxers, Golden Retrievers, and German Shepherds, regardless of geography. A study in Greater Porto, Portugal, compared NHL in humans and cL in dogs, revealing similarities in geographic distribution and environmental influences [21]. Key findings included gender and age differences at diagnosis, with hormonal factors and neutering trends impacting canine NHL. Large and giant dog breeds were more affected, with a higher prevalence of T-cell lymphoma. Strong spatial correlations between human and canine NHL cases, especially in urban areas, suggested that dogs may act as sentinels for environmental risks [37]. Another study investigated the spatial distribution of lymphoma cases among dogs in the United Kingdom and examined potential environmental risk factors contributing to the disease [23]. The findings suggested a heterogeneous distribution of lymphoma, indicating geographic variation that may be associated with environmental influences, for example herbicide exposure, providing further evidence supporting the role of environmental factors in the development of lymphoma in dogs [38].

In canines, it has been noted that cL occurs more frequently in males, but this was based on anecdotal sources, later confirmed by data from the Veterinary Medical Database, one of the most extensive and comprehensive veterinary databases [33]. It has been observed that the difference in the incidence of cL between sexes decreases in older dogs. Based on this, a theory has been proposed that it may be related to hormonal changes occurring in spayed females similar to those occurring in women reaching menopause [33]. It can be hypothesized that in dogs, sex influences the risk of lymphoma, and the hormone levels in intact females reduce this risk [39], but this requires confirmation in further studies. The mentioned database lacks a division into lymphoma types, so it is not known how many of these cases concern the non-Hodgkin’s type [33]. The possibility of a protective role of endogenous estrogens in the etiology of NHL in this species should be investigated.

## 3. The Molecular Mechanisms of Canine and Human Lymphoma

Canine and human lymphomas share several molecular mechanisms, although there are distinct differences as well. Both types of lymphomas involve genetic and epigenetic alterations that drive the onset and progression of the disease. Key pathways implicated in the pathogenesis of canine lymphoma include the nuclear factor kappa B (NF-κB) pathway, which is often activated in B-cell lymphomas, and the mechanistic target of rapamycin (mTOR) pathway, which is frequently altered in T-cell lymphomas [40,41,42]. Additionally, mutations in genes such as MAP3K14, RB1, TP53, and CDKN2A/B have been identified as contributing factors [41]. NF-κB overactivation in canine lymphoma leads to the dysregulation of genes involved in cell proliferation, apoptosis, and inflammation, contributing to tumorigenesis [43].

Similarly, in human lymphoma, the NF-κB pathway and the PI3K/AKT/mTOR pathway play crucial roles, with genetic mutations in genes such as BCL2, MYC, and TP53 being commonly observed and contributing to the dysregulation of apoptosis and cell cycle control [44,45]. Epigenetic modifications, including DNA methylation and histone acetylation, further influence gene expression and tumor behavior in both canine and human lymphomas [41,45]. Additionally, the tumor microenvironment, comprising immune cells, stromal cells, and extracellular matrix components, significantly impacts lymphoma pathogenesis in both species by providing survival signals and promoting immune evasion [42,45]. Comparative genomic studies have revealed significant similarities between canine and human lymphomas, suggesting that insights gained from canine models could enhance our understanding of human lymphomas and improve therapeutic strategies [41,44].

## 4. Diagnosis of the Lymphomas

### 4.1. Clinical Symptoms

Lymphadenopathy in the neck and head is the most common symptom of NHL among human patients, which usually manifests as an enlarged, painless lymph node, and the disease then spreads to non-adjacent nodes; mediastinal involvement is rare, while abdominal involvement is more common [46]. For extranodal lymphomas, the most common location is the palatine tonsils, which causes patients to experience dysphagia and sore throat, and nasal obstruction if the nasopharynx is involved [46,47]. Lymphoma can be also suspected in general practice, among patients with B-symptoms: fevers, night sweats, and weight loss [48]. Other signs seen in lymphomas include sweet syndrome and dermatologic manifestations; sweet syndrome is a group of symptoms consisting of sudden erythematous skin lesions, fever, leukocytosis, and neutrophilia [49]. In blood tests may be observed neutropenia, severe anemia, thrombocytopenia, and hyperphosphatemia with a >26% increase from baseline [48].

**Figure 1:**
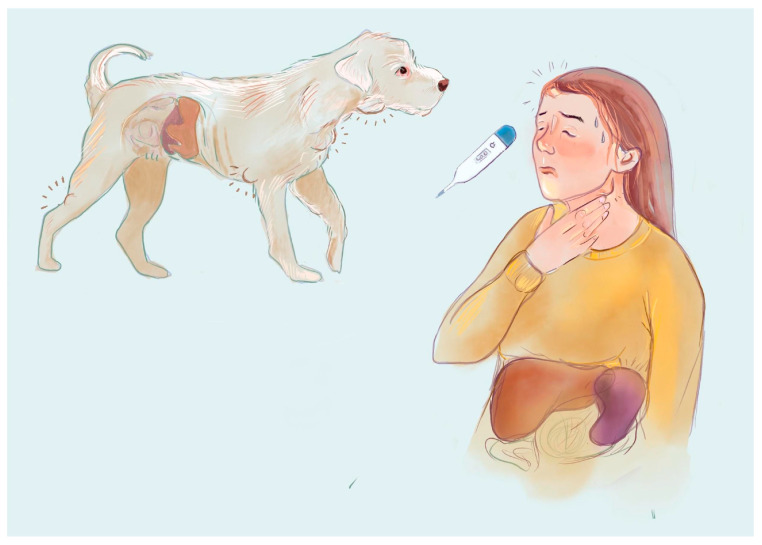
Clinical symptoms of lymphomas. Symptoms of lymphoma in dogs may include general lymphadenopathy, uveitis, weight loss, cranial vena syndrome, and enlargement of spleen and liver, leading to discomfort in the abdomen [19]. Human patients may suffer from single lymph node enlargement, abdominal pain caused by splenic and hepatic enlargement, and B-symptoms like fever and night sweats [48].

In dogs, the most common form of cL is the multifocal form, which attacks the peripheral lymph nodes, leading to lymphadenopathy, but mediastinal or abdominal lymphomas are also noted [19]. Symptoms that can be seen in dogs, in addition to lymphadenopathy, are cranial vena syndrome (symmetric, nonpainful, pitting edema of the head, neck, and forelimbs) or uveitis [19]. Blood tests can show the most common paraneoplastic syndrome, hypercalcemia, most often linked precisely to the presence of lymphoma [50] and resulting from the production of PTH-related peptides (PTH-rPs) by T-cell lymphoblasts [51]. Anemia is a frequently encountered abnormality in lymphoma, mostly normocytic and normochromic, together with leukocytosis and thrombocytopenia [52]. A comparison of the characteristic clinical signs of lymphomas in humans and dogs is shown in Figure 1.

### 4.2. Lymphomas Morphology

**Figure 2:**
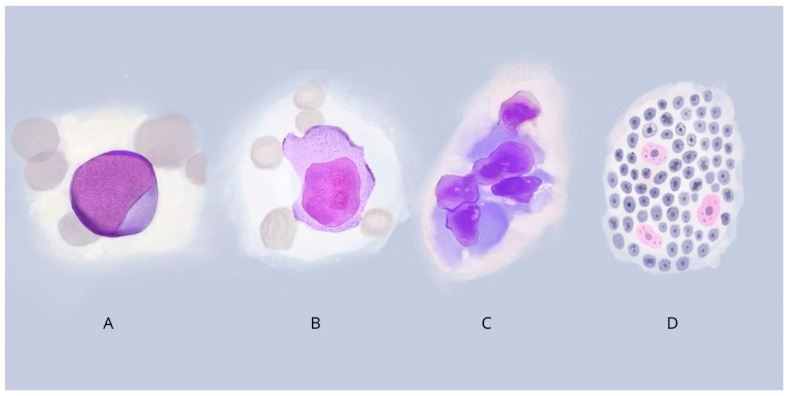
Morphological variations of cells in DLBCL and BL: (**A**) Centroblastic lymphoma: medium to large cell with nuclei containing fine chromatin; (**B**) Immunoblastic lymphoma: cell with a large and centrally located nucleus, with a basophilic cytoplasm; (**C**) Anaplastic lymphoma: cells with large pleomorphic nuclei, resembling Hodgkin/Reed–Sternberg or ALCL cells; (**D**) Burkitt’s lymphoma: The starry sky pattern refers to a monotonous background of basophilic lymphoma cells (sky) within which pale or clear tingible macrophages (stars).

The morphology of DLBCL (diffuse large B-cell lymphoma) in humans is divided into three most common variants: centroblastic, immunoblastic, and anaplastic [53]. Centroblastic lymphoma is characterized by lymphoid cells of medium to large size, with nuclei containing fine chromatin, and it is also the most common type of DLBCL [53]. The immunoblastic variant is a tumor composed of more than 90% immunoblasts, with a large nucleus, a centrally located nucleolus, and a strongly basophilic cytoplasm [53]. In anaplastic lymphoma, on the other hand, we observe cells with large pleomorphic nuclei (one to several per cell) that resemble Hodgkin/Reed–Sternberg cells or anaplastic large cell lymphoma (ALCL) tumor cells [53]. Human Burkitt’s lymphoma (BL) is characterized by medium-sized cells with round nuclei, numerous nucleoli, a basophilic cytoplasm, and frequent mitosis, creating a “starry sky” image [53]. The morphological types of DLBCL and BL cells are shown in Figure 2.

**Figure 3:**
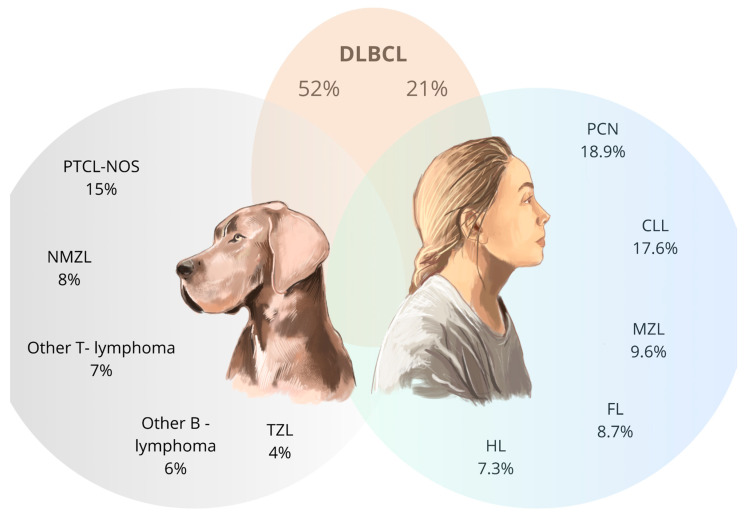
Comparison of the incidence of the most common varieties of lymphoma in humans and dogs (Lamb et al., 2024) Legend: DLBCL—diffuse large B-cell lymphoma, PTCL-NOS—peripheral T-cell lymphoma, not otherwise specified, NMZL—nodal marginal zone lymphoma, TZL—T-zone lymphoma, HL—Hodgkin’s lymphoma, FL—follicular lymphoma, MZL—marginal zone lymphoma, CLL—chronic lymphocytic leukemia, PCN—plasma cell neoplasm. The blue circle represents lymphomas common in human patients, the grey circle those typical for dogs, and the beige circle the one shared by both species [3,56].

Morphologically, canine lymphomas are similar to human DLBCL [54]. In their evaluation, tumor architecture, tumor cell morphology (i.e., size, shape and nuclear/cytoplasmic features) and mitotic activity are considered [3]. The most common types of lymphomas described in dogs were also centroblastic and immunoblastic variants, whose morphological classification is similar to that used in humans, and described in the REAL (Revised European-American Classification of Lymphoid Neoplasms) and WHO classification [55]. In dogs, as in humans, DLBCL tumors consist of large B lymphocytes that obliterate normal tissue morphology, appearing nodal and extranodal, particularly intestinal and mucosal (MALT) [55]. Macronuclear medium-sized cells (MMCs), mixed with large blastic cells, are commonly observed in dogs for polymorphonuclear centroblastic lymphoma and immunoblastic lymphoma, but have not yet been observed in humans, suggesting a transformation of these lymphoma types in dogs from marginal zone lymphoma [55]. Other common morphological types of lymphoma in dogs are peripheral T-cell lymphoma (PTCL-NOS), nodal marginal zone lymphoma (NMZL), and T-zone lymphoma (TZL), which are relatively less common in humans [3]. Tumors similar to Burkitt’s lymphoma in dogs are morphologically similar to those in humans, and in the case of these tumors in the canine population, an additional high growth fraction (80% of cells positive for Ki-67) has been observed, which is similar to the nearly 100% Ki-67 value in Burkitt’s lymphoma in human patients [55]. Table 1 presents different forms and histologic characteristics of cL and NHL lymphoma. At the same time, in Figure 3, there is a comparison of incidence among the most common histological forms of lymphoma in dogs and humans.

## 5. Treatment

### 5.1. Staging

**Figure 4:**
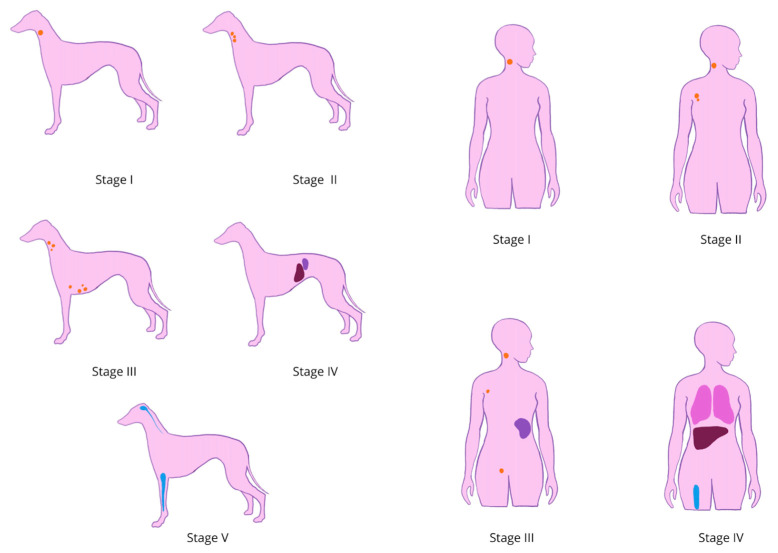
Graphic comparison of WHO staging system for lymphoma in dogs and Lugano modification of Ann Arbor staging systems of lymphomas in humans. In dogs: Stage I—Involvement limited to a single node or lymphoid tissue in a single organ (excluding bone marrow); Stage II—Involvement of many lymph nodes in a regional area (with or without involvement of the tonsils); Stage III—Generalized lymph node involvement; Stage IV—Stage I, II, or III with liver or spleen involvement; Stage V—Stage I, II, III, or IV with manifestation in the blood and involvement of bone marrow, OUN, or other organ systems. In humans: Stage I: Involvement in 1 lymph region only/single extranodal site; Stage II: Involvement in ≥2 lymph regions on the same side of the diaphragm, and may include limited contiguous extranodal involvement; Stage III: In the lymph nodes, spleen, or both and on both sides of the diaphragm; Stage IV: Extranodal involvement (bone marrow, lungs, liver).

Treatment of the disease depends on the clinical staging of the lymphoma, which furthermore helps with defining disease location, provides prognostic information, and allows comparison among studies [57]. Classification of lymphoma in humans in clinical practice is based on the Lugano modification of the Ann Arbor scale, distinguishing four stages of the disease and an E subclassification, denoting involvement of a single extranodal area in stage I or limited involvement of adjacent nodal areas in stage II [58]. It also defines bulky disease, which is an extensive nodal mass defined as greater than 5–10 cm, visible on CT [59]. Staging in dogs is a form of WHO staging, also taking into account the presence of systemic symptoms [19]. A comparison of these staging systems is explained in Table 2 and illustrated in Figure 4.

### 5.2. Chemotherapy Protocols

The treatment of cL primarily revolves around chemotherapy, which is the most effective approach, particularly for the multicentric form of the disease [40]. The CHOP protocol (cyclophosphamide, doxorubicin, vincristine, and prednisone) is the standard regimen, utilizing a combination of drugs to target cancer cells from multiple angles, reduce resistance, and minimize side effects [40]. Another safe and effective method for the treatment for lymphoma in dogs is rabacfosadine (RAB), a novel chemotherapy agent conditionally approved for the treatment of lymphoma in dogs [60]. RAB is a chemotherapy drug developed specifically for dogs, and, unlike standard chemotherapy drugs, RAB has a unique mechanism of action, making it suitable for use in combination with other treatments or in CHOP-based protocols to extend remission and delay drug resistance [60]. A new, first conditionally approved oral drug to treat lymphoma in dogs is verdinexor in tablets [61]. It shows potential in maintaining or improving dogs’ quality of life, though its role in oncology protocols is still being studied. Current research focuses on optimizing dosage, identifying chemotherapy combinations, and determining its best applications [62]. Chemotherapy is typically administered in cycles over several months and, while it may cause side effects like vomiting, appetite loss, or temporary hair thinning, it is often well-tolerated by dogs and can lead to remission lasting up to a year or more [40]. Commonly included in treatment of cL are steroids, such as prednisone, for their anti-inflammatory and immune-modulating effects. They prove to be particularly effective in reducing lymph node swelling and are also used for palliative care in cases where chemotherapy is not feasible. However, steroids alone are not curative and are usually part of a broader treatment plan [63].

Another option for localized lymphoma cases is radiation therapy, as it targets specific lymph nodes or affected areas. Though less commonly used for the multicentric type, it can be beneficial in certain scenarios [64]. Dogs undergoing radiation therapy often require multiple anesthesia sessions due to the need for precise targeting. An experimental approach is bone marrow transplantation, and while potentially groundbreaking, it is not yet a standard practice due to its high cost and complexity [65]. Other emerging treatments are immunotherapy and targeted therapy, such as monoclonal antibodies that selectively target cancer cells. Notable examples include Anti-CD20, mAb 231, and Anti-HLA-DR [66]. For advanced cases or when aggressive treatment is not an option, palliative care prioritizes comfort and quality of life through symptom management and pain relief. This approach ensures that even in untreatable stages, the dog’s well-being remains the central focus.

Lymphoma treatment in humans involves a multifaceted approach tailored to the type and stage of the disease. Chemotherapy is the cornerstone treatment, with regimens like CHOP or R-CHOP commonly used for aggressive NHL and ABVD for Hodgkin’s lymphoma [67]. R-CHOP combines rituximab (a monoclonal antibody targeting CD20) with cyclophosphamide, doxorubicin, vincristine, and prednisone, while ABVD stands for a treatment with doxorubicin, bleomycin, vinblastine, and dacarbazine [68]. Radiation therapy is employed to treat localized lymphoma, particularly in Hodgkin’s lymphoma, where it helps shrink tumors in specific areas. In advanced stages, it complements chemotherapy to target residual disease [69]. For relapsed or refractory cases, stem cell or bone marrow transplants may be considered. These involve either harvesting the patient’s stem cells (autologous) or using a donor’s (allogeneic) after intensive chemotherapy to rebuild the immune system. While potentially curative, these procedures carry significant risks, including infection and graft-versus-host disease [70]. Immunotherapy is also a rapidly advancing field. Monoclonal antibodies like rituximab and chimeric antigen receptor T-cell therapy (CAR T-cell therapy), which genetically modifies a patient’s T cells to target lymphoma cells, are showing promise in treating aggressive lymphomas [71]. Additionally, new targeted drugs, such as Bruton’s tyrosine kinase (BTK) inhibitors and phosphoinositide 3-kinase (PI3K) inhibitors, focus on disrupting molecular pathways that are crucial for lymphoma cell survival [72]. Targeted therapy provides a more personalized treatment approach, aiming at specific cancer growth molecules and offering fewer side effects compared to traditional chemotherapy [72]. For patients with advanced lymphoma, palliative care focuses on improving quality of life through symptom management, pain control, and emotional support.

Assessing prognosis in human patients with DLBCL (diffuse large B-cell lymphoma) is usually based on risk assessment using the International Prognostic Index (IPI), which takes into account the following: age over 60, elevated serum lactate dehydrogenase (LDH) levels, advanced-stage disease, or poor performance status as poor prognostic factors [73]. In the case of lobular lymphoma, patients’ survival time can exceed 10 years, and it is a disease with a variable course, with stage I and II patients usually having a benign disease course [73]. Mantle cell lymphoma has a similar presentation, with a median survival of about 5 years, and a certain group of patients may only be observed at first [73]. An increase in LDH activity is an important prognostic factor in human NHL [74]; however, this test has not been shown to be useful in a similar evaluation in dogs with cL [75].

Response to therapy in dogs also depends on the type and subtype of lymphoma [2]. Complete remission is more often achieved in animals with aggressive B-cell lymphoma than in other morphological types. Indolent lymphomas, in general, do not respond to chemotherapy, and even if remission is achieved, the disease almost always returns [76]. Among them, marginal zone lymphoma, mantle cell lymphoma, and T-cell lymphoma are distinguished. In contrast to subtype-independent survival times of 10–12 months for canine B-cell lymphoma and 6 months for canine T-cell lymphoma, subtype-specific survival times of 21 and 33 months have been reported for indolent forms of lymphoma [3]. At the same time, indolent lymphomas do not always require treatment and can often be treated as a chronic disease that requires only “watchful waiting” [77]. Aggressive T-cell lymphomas, on the other hand, are considered cancers with a low response rate to treatment, and thus a poorer prognosis and incidence of relapse, and CHOP-based protocols in dogs have failed to induce sustained remission in most dogs with aggressive T-cell lymphomas [76].

### 5.3. Molecular Targeted Therapy

Molecular targeted therapy has revolutionized the treatment of both human and canine lymphoma by focusing on specific molecular pathways involved in tumorigenesis. In human lymphoma, targeted therapies such as rituximab, brentuximab vedotin, and CAR T-cell therapy have shown significant efficacy by targeting surface antigens and intracellular pathways, including the NF-κB and PI3K/AKT/mTOR pathways [78,79]. These therapies have improved response rates and overall survival, although resistance mechanisms such as antigen escape and mutations in signaling pathways remain challenges [78]. In canine lymphoma, targeted therapies are still in the early stages of development, but optimistic results have been observed with agents targeting similar pathways, such as the NF-κB and mTOR pathways [40,66]. Inhibitors of the NF-κB pathway, such as parthenolide, have shown promise in preclinical studies by inducing apoptosis and reducing tumor growth in canine lymphoma models [42].

Comparative studies have highlighted the potential of using canine models to bridge the translational gap between preclinical studies and human clinical trials, given the similarities in disease biology and response to therapy [66]. Further research into these targeted therapies could lead to more effective and personalized treatment options for canine lymphoma, ultimately improving prognosis and survival rates [40]. Both human and canine lymphomas benefit from these therapies, which disrupt key molecular pathways, but the development and application of these therapies are more advanced in human medicine [66,78]. The main differences lie in the availability and regulatory approval of targeted agents, with more options currently approved for human use [79].

### 5.4. The Influence of Antiestrogens

The beneficial effects of antiestrogens (such as tamoxifen or diethylstilbestrol) have been suggested in the remission of chronic lymphocytic leukemia and recurrent lymphomas in humans [80]. However, in dogs, estrogen and progesterone receptors are practically undetectable in the cytosol of cancer cells [80], indicating the ineffectiveness of hormone therapy in this species. This was confirmed in a pilot study on five dogs with NHL, where despite the presence of numerous binding sites for antiestrogens, no positive response to treatment was observed [80].

Tumor-suppressing effects of sex hormones on mantle cell lymphoma have been observed in a recent study, where mice were transplanted with MCL cells and treated with estrogen, selectively activating its receptor β (ESR2). The activation of ESR2 led to a decrease in MCL tumor growth, potentially explaining the observed sex differences in mankind. Additionally, targeting ESR2 could be considered as a treatment option for MCL [81]. Both selective and non-selective estrogen receptor β (ERβ) agonists exhibit antiproliferative and pro-apoptotic effects on human lymphoma cell lines, suggesting their potential efficacy in treating ERβ-positive lymphomas and leukemias. However, in canine lymphoma tissues, estrogen receptor (ER) expression has been barely noticed over the years. Preliminary studies, based on available data, indicate that unlike in humans, the predominant ER in canine hematopoietic tumors is ERα [82].

## 6. Can a Dog Be a Model for Lymphoma in Humans?

The primary animal models for lymphoid tumor research are usually immunocompromised mice with transplanted human tumor cells or mice with a disabled gene whose blockade makes it possible to study oncogenic pathways [1]. In laboratory studies, however, immunocompetent mice, in which tumor induction and growth prove problematic, are an obstacle. Gene-excluded mice, suitable for monogenic diseases, may also be a difficulty, which may not be sufficient for polygenic lymphoma research [1]. The canine model can overcome some of the stated difficulties in conducting in-vivo studies. The incidence of lymphoma in dogs is spontaneous, involving animals without a homogeneous genetic background, as well as immunocompetent patients. Studies can be conducted without genetic engineering. It is also possible to organize dogs into breeds with a relatively homogeneous genetic background and a clear predisposition to lymphoma [5].

Due to common risk factors and similar developmental pathways, as well as histopathological and biological features, cL can be considered a potential model for NHL research in humans [5]. This observation lays the groundwork for revolutionary research that could accelerate advances in the treatment of these cancers, which are occurring with increasing frequency in populations of both species [19]. In addition to the reported increase in incidence in both species, there are many other similarities between cL and NHL, including clinical presentation, molecular biology, treatment course, and response to therapy [19]. Dogs can, therefore, serve as models for research into the causes, progression, and development of new therapies for lymphoma. The similarities between cL and NHL make the dog a suitable model for research for both veterinary medicine and human medicine. Translational medicine makes the development of research about cL beneficial for human patients, as NHL accounts for nearly 3% of cancer diagnoses and deaths, which makes it the seventh most prevalent cancer, and its incidence has increased 168% since 1975 [9].

Analyzing the effect of the environment on the development of lymphoma in dogs can provide valuable insights into how the same environment influences the incidence of this disease in humans. The shorter lifespan of dogs means that these observations can be made much more quickly than in humans [4]. A canine model of lymphoma was also used to evaluate the usefulness of an anti-HLA-DR monoclonal antibody (mAb), which can help achieve disease stabilization in both humans and dogs [83]. Moreover, the findings supported the idea that the canine model could be used to evaluate the safety and efficacy of the anti-HLA-DR mAb in both dogs and humans [83]. The similarities between canine and human cancers make it possible to study complex immune interactions during immunotherapy [66]. This provides new opportunities for drug discovery that can be used in treatment protocols in both species. However, the further development of immunotherapy requires many efforts, in which the inclusion of the canine model in the path of faster development of immunotherapeutic drugs seems to be an important solution, enabling the emergence of an integrated platform for drug discovery through the collaboration of veterinary and human medicine [84].

Due to the increasing lifespan of dogs, it is possible to observe environmental factors influencing diseases that manifest later in life. Canines, as a more closely related species, enable more precise analysis of drug parameters such as pharmacokinetics and pharmacodynamics [66]. As a result of the shorter development time of lymphoma in dogs, it is more manageable to delve into the topic of tumor initiation and promotion, circumventing certain limitations present in the murine model [1,4]. Consequently, it enables us to incorporate the findings of pet trials in human trials. Eventually, correspondence between lymphomagenesis may help in identifying key gene mutations in both species [5].

## 7. Conclusions

The research of lymphoma in dogs provides a remarkable opportunity for advancing both animal and human medicine. Canines present with lymphomas closely related to human NHLs, sharing not only similar environmental risk factors but also histologic characteristics and spontaneous disease progression, making them a model that closely resembles human lymphomas. This can contribute to a more translational approach in studying lymphoma pathogenesis and progression, as well as therapeutic responses. With advances in molecular therapies, including targeted therapies, immunotherapy, gene therapy, molecular biomarkers, and microbiome research, both species stand to benefit. In particular, the study of lymphoma in dogs offers insight into the efficacy of inhibitors targeting key pathways like NF-κB and PI3K/Akt/mTOR and how immunotherapies such as monoclonal antibodies and CAR-T cell treatments can be adapted for veterinary use. As research progresses, canine models will continue to inform developments in human medicine, helping refine cancer treatments and improve diagnostics. Implementing the One Health approach to researching interactions between human and animal health underscores how advancements in one field can propel progress in the other, making modern medicine more versatile, innovative, and impactful for all species.

## Figures and Tables

**Table 1:** Comparison of the histological structure of lymphomas occurring in dogs and humans [3].

Form of the Lymphoma	Canine—Histologic Characteristics	Human—Histologic Characteristics
DLBCL	Large cells with round nuclei. One or multiple nucleoli. High mitotic rate and “starry sky” appearance.	Large cells with round nuclei. Both immunoblasts and centroblasts. High mitotic rate and “starry sky” appearance.
Mantle Cell Lymphoma	Small- to intermediate-sized cells; scant cytoplasm, round nuclei with dense chromatin, and inconspicuous nucleoli. Varied mitotic rate.	Very heterogeneous; small to large cells; round to irregular nuclei; varied nucleoli and mitotic rate
Splenic Marginal Zone Lymphoma	Intermediate-sized cells; abundant pale cytoplasm; irregular nuclei with peripheralized chromatin, single nucleolus; rare mitotic figures.	Biphasic-Small cells with scant cytoplasm and round nuclei; intermediate-sized cells with abundant cytoplasm and irregular nuclei; rare mitotic figures.
Nodal Marginal Zone Lymphoma	Mixed. Mostly intermediate-sized cells with pale cytoplasm, irregular nuclei, peripheral chromatin, and one nucleolus.	Intermediate-sized cells with pale cytoplasm and irregular nuclei (centrocyte-like) and large cells with abundant pale cytoplasm and irregular nuclei (monocytoid).
Follicular Lymphoma	Mixed. Mostly small cells with clear cytoplasm, pale chromatin, and inconspicuous nucleoli (centrocytes) with fewer large cells with dark blue cytoplasm, vesicular nuclei, and 1–3 nucleoli (centroblasts).	Similar
PTCL-NOS (Peripheral T-cell lymphoma)	Small to large (heterogeneous) with irregular nuclei, variable chromatin, prominent nucleoli, and varied mitotic activity	Similar
T-zone lymphoma	Small- to intermediate-sized cells; moderate amount of pale cytoplasm; oval to elliptical nuclei with sharp, shallow indentations; nucleoli and mitotic figures are sparse.	Similar

**Table 2:** Comparison of Lugano Ann Arbor staging systems in humans and WHO clinical staging system for dogs.

Lugano Modification of Ann Arbor Staging Systems of Lymphomas in Humans	WHO Clinical Staging System for Lymphoma in Dogs
Stage I: Involvement in 1 lymph region only or single extranodal site	Stage I: Involvement limited to a single node or lymphoid tissue in a single organ (excluding bone marrow)
Stage II: Involvement in ≥2 lymph regions on the same side of the diaphragm, and may include limited contiguous extranodal involvement	Stage II: Involvement of many lymph nodes in a regional area (with or without involvement of the tonsils)
Stage III: In the lymph nodes, spleen, or both and on both sides of the diaphragm	Stage III: Generalized lymph node involvement
Stage IV: extranodal involvement (bone marrow, lungs, liver)	Stage IV: Stage I, II, or III with liver or spleen involvement
-	Stage V: Stage I, II, III, or IV with manifestation in the blood and involvement of bone marrow or other organ systems
Subclassification E indicates single extranodal site involvement in stage I or limited contiguous extranodal involvement in stage II. Involvement of non-contiguous extranodal sites is considered stage IV. Bulky disease is defined as a single nodal mass of ≥10 cm in maximum dimension based on CT imaging.	Each stage is further classified into substages based on the presence of systemic signs: substage a = absence of systemic signs; substage b = presence of systemic signs (fever, >10% weight loss, hypercalcemia)

## Abbreviations

ALCL	anaplastic large cell lymphoma
ABVD	chemotherapy protocol based on doxorubicin, bleomycin, vinblastine, and dacarbazine
BL	Burkitt’s lymphoma
BTK	Bruton’s tyrosine kinase
CHOP	chemotherapy protocols based on C-cyclophosphamide, doxorubicin, vincristine, and prednisolone
CL	canine lymphoma
DLBCL	diffuse large B-cell lymphoma
ER	estrogen receptor
ERα	estrogen receptor α
ERβ	estrogen receptor β
ESR2	estrogen’s receptor 2
IPI	International Prognostic Index
LDH	lactate dehydrogenase
MALT	mucosa-associated lymphoid tissue
MCL	mantle cell lymphoma
MMCs	macronuclear medium-sized cells
mAb	monoclonal antibody
mTOR	mechanistic target of rapamycin
NF-κB	nuclear factor kappa B
NHL	non-Hodgkin’s lymphoma
NMZL	nodal marginal zone lymphoma
NK	natural killer
PI3K	phosphoinositide 3-kinase inhibitors
PTH-rP	PTH-related peptide
PTCL-NOS	peripheral T-cell lymphoma
RAB	rabacfosadine
R-CHOP	chemotherapy protocol based on rituximab with cyclophosphamide, doxorubicin, vincristine and prednisone
REAL	Revised European-American Classification of Lymphoid Neoplasm
TZL	T-zone lymphoma
WHO	World Health Organisation

## References

[1] Marconato L., Gelain M.E., Comazzi S. (2012). The Dog as a Possible Animal Model for Human Non-Hodgkin Lymphoma: A Review. Hematol. Oncol..

[2] Valli V.E., Kass P.H., Myint M.S., Scott F. (2013). Canine Lymphomas. Vet. Pathol..

[3] Seelig D., Avery A., Ehrhart E., Linden M. (2016). The Comparative Diagnostic Features of Canine and Human Lymphoma. Vet. Sci..

[4] Wise C.F., Breen M., Stapleton H.M. (2024). Canine on the Couch: The New Canary in the Coal Mine for Environmental Health Research. Environ. Health.

[5] Ito D., Frantz A.M., Modiano J.F. (2014). Canine Lymphoma as a Comparative Model for Human Non-Hodgkin Lymphoma: Recent Progress and Applications. Vet. Immunol. Immunopathol..

[6] Coyle K.M., Hillman T., Cheung M., Grande B.M., Bushell K.R., Arthur S.E., Alcaide M., Thomas N., Dreval K., Wong S. (2022). Shared and Distinct Genetic Features in Human and Canine B-Cell Lymphomas. Blood Adv..

[7] Johnson R.E., Cameron T.P., Kinard R. (1968). Canine lymphoma as a potential model for experimental therapeutics. Cancer Res..

[8] Hushmandi K., Bokaie S., Shirani D., Taghipour A. (2023). Canine Lymphoma as a Possible Human Lymphoma Model: A Case-Series Study. J. Vet. Clin..

[9] Thandra K.C., Barsouk A., Saginala K., Padala S.A., Barsouk A., Rawla P. (2021). Epidemiology of Non-Hodgkin’s Lymphoma. Med. Sci..

[10] National Cancer Institute, Surveillance, Epidemiology, and End Results Program Cancer Stat Facts: Non-Hodgkin Lymphoma. https://seer.cancer.gov/statfacts/html/nhl.html.

[11] Chu Y., Liu Y., Fang X., Jiang Y., Ding M., Ge X., Yuan D., Lu K., Li P., Li Y. (2023). The Epidemiological Patterns of Non-Hodgkin Lymphoma: Global Estimates of Disease Burden, Risk Factors, and Temporal Trends. Front. Oncol..

[12] Liu W., Song Y., Zhu J., Ma J., Ma Z., Yang A. (2023). Introduction of lymphoma database of national health commission of the people’s republic of China. Hematol. Oncol..

[13] Sedeta E., Ilerhunmwuwa N., Wasifuddin M., Uche I., Hakobyan N., Perry J., Aiwuyo H., Abowali H., Avezbakiyev B. (2022). Epidemiology of Non-Hodgkin Lymphoma: Global Patterns of Incidence, Mortality, and Trends. Blood.

[14] Bawa M., Salari Y., Taylor-Gallardo E., Memon A. (2023). Increasing Incidence of Non-Hodgkin Lymphoma in England, 1985–2019. Eur. J. Environ. Public Health.

[15] Bowzyk Al-Naeeb A., Ajithkumar T., Behan S., Hodson D.J. (2018). Non-Hodgkin lymphoma. BMJ.

[16] Luo J., Craver A., Bahl K., Stepniak L., Moore K., King J., Zhang Y., Aschebrook-Kilfoy B. (2022). Etiology of Non-Hodgkin Lymphoma: A Review from Epidemiologic Studies. J. Natl. Cancer Cent..

[17] Perry A.M., Diebold J., Nathwani B.N., MacLennan K.A., Müller-Hermelink H.K., Bast M., Boilesen E., Armitage J.O., Weisenburger D.D. (2016). Non-Hodgkin Lymphoma in the Developing World: Review of 4539 Cases from the International Non-Hodgkin Lymphoma Classification Project. Haematologica.

[18] Ruple A., Avery A.C., Morley P.S. (2016). Differences in the Geographic Distribution of Lymphoma Subtypes in Golden Retrievers in the USA. Vet. Comp. Oncol..

[19] Zandvliet M. (2016). Canine Lymphoma: A Review. Vet. Q..

[20] Messina M.L., Quintavalla F., Giannuzzi A.P., Furlanello T., Caldin M. (2024). An Evaluation of Hemostatic Dysregulation in Canine Multicentric Lymphoma. Animals.

[21] Pinello K.C., Niza-Ribeiro J., Fonseca L., de Matos A.J. (2019). Incidence, characteristics and geographical distributions of canine and human non-Hodgkin’s lymphoma in the Porto region (North West Portugal). Vet. J..

[22] Marconato L., Leo C., Girelli R., Salvi S., Abramo F., Bettini G., Comazzi S., De Nardi P., Albanese F., Zini E. (2009). Association between Waste Management and Cancer in Companion Animals. J. Vet. Intern. Med..

[23] Schofield I., Stevens K.B., Pittaway C., O’Neill D.G., Fecht D., Dobson J.M., Brodbelt D.C. (2019). Geographic Distribution and Environmental Risk Factors of Lymphoma in Dogs under Primary-Care in the UK. J. Small Anim. Pract..

[24] Zanini D.A., Kimura K.C., Nishiya A.T., Ubukata R., Leandro R.M., de Brito C.P., Trombetti M., Lagoa A.C., Macedo T.R., Rodrigues L.C.d.S. (2013). Environmental Risk Factors Related to the Development of Canine Non-Hodgkin’s Lymphoma. Ciênc. Rural.

[25] Gavazza A., Presciuttini S., Barale R., Lubas G., Gugliucci B. (2001). Association between Canine Malignant Lymphoma, Living in Industrial Areas, and Use of Chemicals by Dog Owners. J. Vet. Intern. Med..

[26] Boyle J., Ward M.H., Cerhan J.R., Rothman N., Wheeler D.C. (2023). Modeling Historic Environmental Pollutant Exposures and Non-Hodgkin Lymphoma Risk. Environ. Res..

[27] Odutola M.K., van Leeuwen M.T., Turner J., Bruinsma F., Seymour J.F., Prince H.M., Milliken S.T., Trotman J., Verner E., Tiley C. (2022). Associations between Smoking and Alcohol and Follicular Lymphoma Incidence and Survival: A Family-Based Case-Control Study in Australia. Cancers.

[28] Skibola C.F., Bracci P.M., Halperin E., Nieters A., Hubbard A., Paynter R.A., Skibola D.R., Agana L., Becker N., Tressler P. (2008). Polymorphisms in the Estrogen Receptor 1 and Vitamin c and Matrix Metalloproteinase Gene Families Are Associated with Susceptibility to Lymphoma. PLoS ONE.

[29] Cerhan J.R. (2002). Anthropometric Characteristics, Physical Activity, and Risk of Non-Hodgkin’s Lymphoma Subtypes and B-Cell Chronic Lymphocytic Leukemia: A Prospective Study. Am. J. Epidemiol..

[30] Lee J.S., Bracci P.M., Holly E.A. (2008). Non-Hodgkin Lymphoma in Women: Reproductive Factors and Exogenous Hormone Use. Am. J. Epidemiol.

[31] Kane E.V., Bernstein L., Bracci P.M., Cerhan J.R., Costas L., Dal Maso L., Holly E.A., La Vecchia C., Matsuo K., Sanjose S. (2013). Postmenopausal Hormone Therapy and Non-Hodgkin Lymphoma: A Pooled Analysis of InterLymph Case–Control Studies. Ann. Oncol..

[32] Schneider R., Dorn C.R., Taylor D.O.N. (1969). Factors Influencing Canine Mammary Cancer Development and Postsurgical Survival. J. Natl. Cancer Inst..

[33] Villamil J.A., Henry C.J., Hahn A.W., Bryan J.N., Tyler J.W., Caldwell C.W. (2009). Hormonal and Sex Impact on the Epidemiology of Canine Lymphoma. J. Cancer Epidemiol..

[34] Bennett P.F., Taylor R., Williamson P. (2018). Demographic Risk Factors for Lymphoma in Australian Dogs: 6201 Cases. J. Vet. Intern. Med..

[35] Modiano J.F., Breen M., Burnett R.C., Parker H.G., Inusah S., Thomas R., Avery P.R., Lindblad-Toh K., Ostrander E.A., Cutter G.C. (2005). Distinct B-Cell and T-Cell Lymphoproliferative Disease Prevalence among Dog Breeds Indicates Heritable Risk. Cancer Res..

[36] Atherton M.J., Mason N.J. (2022). A Bitesize Introduction to Canine Hematologic Malignancies. Blood Adv..

[37] Chibuk J., Flory A., Kruglyak K.M., Leibman N., Nahama A., Dharajiya N., van den Boom D., Jensen T.J., Friedman J.S., Shen M.R. (2021). Horizons in Veterinary Precision Oncology: Fundamentals of Cancer Genomics and Applications of Liquid Biopsy for the Detection, Characterization, and Management of Cancer in Dogs. Front. Vet. Sci..

[38] Pinello K., Leite-Martins L., Gregório H., Oliveira F., Kimura K.C., Dagli M.L.Z., de Matos A., Niza-Ribeiro J. (2025). Exploring Risk Factors Linked to Canine Lymphoma: A Case-Control Study. Top. Companion Anim. Med..

[39] Henry C.J., Hahn A.W., Tyler J.W., Steinberg H.S., Caldwell C.W. (2005). Comparative Oncology Solution to Understanding Hormonal Impact in the Epidemiology of Lymphoma. Vet. Comp. Oncol..

[40] Bennett P., Williamson P., Taylor R. (2023). Review of Canine Lymphoma Treated with Chemotherapy-Outcomes and Prognostic Factors. Vet. Sci..

[41] Cao J., Kouznetsova V.L., Tsigelny I.F. (2019). Molecular Mechanisms of Canine Cancers. OBM Genet..

[42] Castiglioni V., Sforna M., de Vries C. (2024). Editorial: Canine Lymphoma Pathogenesis, Diagnosis, Prognosis and Treatment: Current and Future Perspectives. Front. Vet. Sci..

[43] Schlein L.J., Thamm D.H. (2022). Review: NF-KB Activation in Canine Cancer. Vet. Pathol..

[44] Küppers R. (2009). Molecular Biology of Hodgkin Lymphoma. Hematol. Am. Soc. Hematol. Educ. Program..

[45] Cerchietti L. (2024). Genetic Mechanisms Underlying Tumor Microenvironment Composition and Function in Diffuse Large B-Cell Lymphoma. Blood.

[46] Singh R., Shaik S., Negi B.S., Rajguru J.P., Patil P.B., Parihar A.S., Sharma U. (2020). Non-Hodgkin’s Lymphoma: A Review. J. Fam. Med. Prim. Care.

[47] Walter C., Ziebart T., Sagheb K., Rahimi-Nedjat R.K., Manz A., Hess G. (2015). Malignant Lymphomas in the Head and Neck Region—A Retrospective, Single-Center Study over 41 Years. Int. J. Med. Sci..

[48] Paquin A.R., Oyogoa E., McMurry H.S., Kartika T., West M., Shatzel J.J. (2022). The Diagnosis and Management of Suspected Lymphoma in General Practice. Eur. J. Haematol..

[49] Agrawal A., Arif S.H., Kumarasan K., Janjua D. (2022). Sweet’s Syndrome: An Update. Curr. Pediatr. Rev..

[50] Messinger J.S., Windham W.R., Ward C.R. (2009). Ionized Hypercalcemia in Dogs: A Retrospective Study of 109 Cases (1998–2003). J. Vet. Intern. Med..

[51] Ruslander D.A., Gebhard D.H., Tompkins M.B., Grindem C.B., Page R.L. (1997). Immunophenotypic Characterization of Canine Lymphoproliferative Disorders. PubMed.

[52] Thangapandiyan M., Balachandran C., Jeyaraja K., Raja A., Sridhar R. (2017). Study on Haematological Alterations in Canine Lymphoma. Deleted J..

[53] Chen B.-J., Fend F., Campo E., Quintanilla-Martinez L. (2019). Aggressive B-Cell Lymphomas—From Morphology to Molecular Pathogenesis. Ann. Lymphoma.

[54] Richards K.L., Motsinger-Reif A., Chen H.-W., Fedoriw Y.D., Fan C., Nielsen D.M., Thomas R., Smith C., Dave S.S., Perou C.M. (2011). Characterizing Canine Lymphoma as a Potential Large Animal Model of Human Diffuse Large B-Cell Lymphoma. Blood.

[55] Ponce F., Marchal T., Magnol J.P., Turinelli V., Ledieu D., Bonnefont C., Pastor M., Delignette M.L., Fournel-Fleury C. (2010). A Morphological Study of 608 Cases of Canine Malignant Lymphoma in France with a Focus on Comparative Similarities between Canine and Human Lymphoma Morphology. Vet. Pathol..

[56] Lamb M., Painter D., Howell D., Barrans S., Cargo C., de Tute R., Tooze R., Burton C., Patmore R., Roman E. (2024). Lymphoid Blood Cancers, Incidence and Survival 2005-2023: A Report from the UK’s Haematological Malignancy Research Network. Cancer Epidemiol..

[57] Cheson B.D., Fisher R.I., Barrington S.F., Cavalli F., Schwartz L.H., Zucca E., Lister T.A. (2014). Recommendations for Initial Evaluation, Staging, and Response Assessment of Hodgkin and Non-Hodgkin Lymphoma: The Lugano Classification. J. Clin. Oncol..

[58] Cheson B.D. (2015). Staging and Response Assessment in Lymphomas: The New Lugano Classification. Chin. Clin. Oncol..

[59] Ma’koseh M., Farfoura H., Khatib Y., Omari Z., Ababneh H., Fayoumi B.A., Taqash A., Al-Rwashdeh M., Abufara A., Shahin O. (2023). Definition of Bulky Disease in Early Stage Diffuse Large B-Cell Lymphoma in Computed Tomography on Coronal and Transverse Planes. Front. Oncol..

[60] Weishaar K.M., Wright Z.M., Rosenberg M.P., Post G.S., McDaniel J.A., Clifford C.A., Phillips B.S., Bergman P.J., Randall E.K., Avery A.C. (2021). Multicenter, Randomized, Double-Blinded, Placebo-Controlled Study of Rabacfosadine in Dogs with Lymphoma. J. Vet. Intern. Med..

[61] Mealey K.L., Burke N.S. (2023). Assessment of Verdinexor as a Canine P-Glycoprotein Substrate. J. Vet. Pharmacol. Ther..

[62] Sadowski A.R., Gardner H.L., Borgatti A., Wilson H., Vail D.M., Lachowicz J., Manley C., Turner A., Klein M.K., Waite A. (2018). Phase II Study of the Oral Selective Inhibitor of Nuclear Export (SINE) KPT-335 (Verdinexor) in Dogs with Lymphoma. BMC Vet. Res..

[63] Rassnick K.M., Bailey D.B., Kamstock D.A., LeBlanc C.J., Berger E.P., Flory A.B., Kiselow M.A., Intile J.L., Malone E.K., Regan R.C. (2021). Survival Time for Dogs with Previously Untreated, Peripheral Nodal, Intermediate- or Large-Cell Lymphoma Treated with Prednisone Alone: The Canine Lymphoma Steroid Only Trial. J. Vet. Med. Educ..

[64] Best M.P., Straw R.C., Gumpel E., Fry D.R. (2023). Long-Term Remission and Survival in Dogs with High-Grade, B Cell Lymphoma Treated with Chemotherapy with or without Sequential Low-Dose Rate Half-Body Irradiation. J. Vet. Intern. Med..

[65] Graves S.S., Storb R. (2021). Evolution of Haematopoietic Cell Transplantation for Canine Blood Disorders and a Platform for Solid Organ Transplantation. Vet. Med. Sci..

[66] Dias J.N.R., André A.S., Aguiar S.I., Gil S., Tavares L., Aires-da-Silva F. (2021). Immunotherapeutic Strategies for Canine Lymphoma: Changing the Odds against Non-Hodgkin Lymphoma. Front. Vet. Sci..

[67] Major A., Smith S.M. (2021). DA-R-EPOCH vs R-CHOP in DLBCL: How Do We Choose?. Clin. Adv. Hematol. Oncol..

[68] Strati P., Fanale M.A., Oki Y., Turturro F., Fayad L.E., Bartlett N.L., Gladstone D.E., Kasamon Y.L., Portlock C.S., Wilson W.H. (2018). ABVD plus Rituximab *Versus* ABVD Alone for Advanced Stage, High-Risk Classical Hodgkin Lymphoma: A Randomized Phase 2 Study. Haematologica.

[69] Waldstein C. (2022). Radiotherapy Update: Current Role of Radiotherapy in the Treatment of Lymphomas. Memo.

[70] Zahid U., Akbar F., Amaraneni A., Husnain M., Chan O., Riaz I.B., McBride A., Iftikhar A., Anwer F. (2017). A Review of Autologous Stem Cell Transplantation in Lymphoma. Curr. Hematol. Malig. Rep..

[71] Denlinger N., Bond D., Jaglowski S. (2021). CAR T-Cell Therapy for B-Cell Lymphoma. Curr. Probl. Cancer.

[72] Burger J.A. (2019). Bruton Tyrosine Kinase Inhibitors. Cancer J..

[73] Ansell S.M. (2015). Non-Hodgkin Lymphoma: Diagnosis and Treatment. Mayo Clin. Proc..

[74] William B.M., Bongu N.R., Bast M., Bociek R.G., Bierman P.J., Vose J.M., Armitage J.O. (2013). The Utility of Lactate Dehydrogenase in the Follow up of Patients with Diffuse Large B-Cell Lymphoma. Rev. Bras. Hematol. Hemoter..

[75] Marconato L., Crispino G., Finotello R., Mazzotti S., Salerni F., Zini E. (2009). Serum Lactate Dehydrogenase Activity in Canine Malignancies. Vet. Comp. Oncol..

[76] Aresu L., Ferraresso S., Marconato L., Cascione L., Napoli S., Gaudio E., Kwee I., Tarantelli C., Testa A., Maniaci C. (2019). New Molecular and Therapeutic Insights into Canine Diffuse Large B-Cell Lymphoma Elucidates the Role of the Dog as a Model for Human Disease. Haematologica.

[77] Moore A.S. (2016). Treatment of T Cell Lymphoma in Dogs. Vet. Rec..

[78] Deshpande A., Munoz J. (2022). Targeted and Cellular Therapies in Lymphoma: Mechanisms of Escape and Innovative Strategies. Front. Oncol..

[79] D’Alò F., Bellesi S., Maiolo E., Alma E., Bellisario F., Malafronte R., Viscovo M., Campana F., Hohaus S. (2024). Novel Targets and Advanced Therapies in Diffuse Large B Cell Lymphomas. Cancers.

[80] Teske E., Besselink C.M.L.T. (1987). The Occurrence of Estrogen and Progestin Receptors and Anti-Estrogen Binding Sites (AEBS) in Canice Non-Hodgkin’s Lymphomas. Anticancer Res..

[81] Huang D., Huang Z., Indukuri R., Chandrashekar Bangalore R., Berglund M., Guan J., Yakimchuk K., Damdimopoulos A., Williams C., Okret S. (2022). Estrogen Receptor β (ESR2) Transcriptome and Chromatin Binding in a Mantle Cell Lymphoma Tumor Model Reveal the Tumor-Suppressing Mechanisms of Estrogens. Cancers.

[82] Bugiel-Stabla K., Agnoli C., Pawlak A. (2024). Estrogen Receptors Alpha and Beta Expression in Different Canine Cancer Types with an Emphasis on Hematopoietic Malignancies. Vet. Res. Commun..

[83] Stein R., Balkman C., Chen S., Rassnick K., Mcentee M., Page R., Goldenberg D.M. (2010). Evaluation of Anti-Human Leukocyte Antigen-DR Monoclonal Antibody Therapy in Spontaneous Canine Lymphoma. Leuk. Lymphoma.

[84] Decker W.K., da Silva R.F., Sanabria M.H., Angelo L.S., Guimarães F., Burt B.M., Kheradmand F., Paust S. (2017). Cancer Immunotherapy: Historical Perspective of a Clinical Revolution and Emerging Preclinical Animal Models. Front. Immunol..

