# Correction: Będkowska et al. What We Know and Do Not Yet Know About the Canine Model of Lymphoma in Human Medicine—The Current State of Knowledge. *Cancers* 2025, *17*, 596

**DOI:** 10.3390/cancers17091590

**Published:** 2025-05-07

**Authors:** Daria Będkowska, Sara Al-Ameri, Agnieszka Wieczorek, Joanna Bubak, Marta Miszczak

**Affiliations:** 1 EZA Student Science Club, Department of Epizootiology and Clinic of Birds and Exotic Animals, Division of Infectious Diseases and Veterinary Administration, The Faculty of Veterinary Medicine, Wrocław University of Environmental and Life Sciences, Grunwaldzki Sq. 45, 50-366 Wrocław, Poland; 118245@student.upwr.edu.pl (D.B.); 118225@student.upwr.edu.pl (S.A.-A.); 115786@student.upwr.edu.pl (A.W.); 2 Department of Pathology, Division of Pathomorphology and Veterinary Forensics, The Faculty of Veterinary Medicine in Wrocław, Wrocław University of Environmental and Life Sciences, 31 Norwida St., 50-375 Wrocław, Poland; joanna.bubak@upwr.edu.pl; 3 Department of Epizootiology and Clinic of Birds and Exotic Animals, Division of Infectious Diseases and Veterinary Administration, The Faculty of Veterinary Medicine in Wroclaw, Wrocław University of Environmental and Life Sciences, Grunwaldzki Sq. 45, 50-366 Wrocław, Poland

## Error in Figure

In the original publication [1], there was a mistake in Figure 2D as published. Figure 2D represents mitotic figure, but not “Burkitt’s lymphoma”: medium-sized cell with round nuclei and frequent mitosis, creating a “starry sky” image. The corrected Figure 2 appears below.

The authors state that the scientific conclusions are unaffected. This correction was approved by the Academic Editor. The original publication has also been updated.

## Figures and Tables

**Figure 2:**
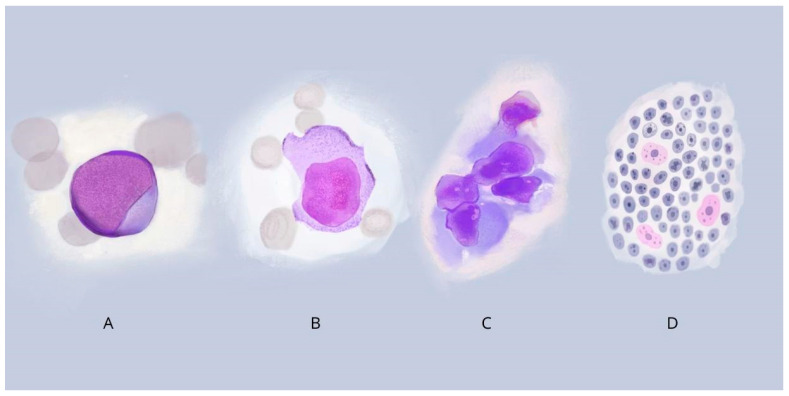
Morphological variations of cells in DLBCL and BL: (**A**) Centroblastic lymphoma: medium to large cell with nuclei containing fine chromatin; (**B**) Immunoblastic lymphoma: cell with a large and centrally located nucleus, with a basophilic cytoplasm; (**C**) Anaplastic lymphoma: cells with large pleomorphic nuclei, resembling Hodgkin/Reed–Sternberg or ALCL cells; (**D**) Burkitt’s lymphoma: The starry sky pattern refers to a monotonous background of basophilic lymphoma cells (sky) within which pale or clear tingible macrophages (stars).
